# Polyamine Putrescine Regulates Oxidative Stress and Autophagy of Hemocytes Induced by Lipopolysaccharides in Pearl Oyster *Pinctada fucata martensii*

**DOI:** 10.3389/fphys.2021.781324

**Published:** 2021-12-10

**Authors:** Yanfei Cao, Yu Jiao, Shuzhi Zhan, Xueru Liang, Zhixin Li, Jiayi Chen, Xinwei Xiong, Zefeng Gu, Xiaodong Du, Zhe Zheng

**Affiliations:** ^1^Fishery College, Guangdong Ocean University, Zhanjiang, China; ^2^Pearl Breeding and Processing Engineering Technology Research Centre of Guangdong Province, Zhanjiang, China; ^3^Guangdong Science and Innovation Center for Pearl Culture, Zhanjiang, China; ^4^Guangdong Provincial Engineering Laboratory for Mariculture Organism Breeding, Zhanjiang, China

**Keywords:** polyamines, putrescine, immune regulation, oxidative stress, mollusks, pearl oysters

## Abstract

The polyamine putrescine (Put) is a ubiquitous small cationic amine. It plays an essential role in controlling the innate immune response. However, little is known about its function in mollusks. In this study, the Put content was observed to increase in the serum of pearl oyster *Pinctada fucata martensii* after 6 and 24 h of lipopolysaccharide (LPS) stimulation. Activities of superoxide dismutase (SOD), catalase (CAT), and glutathione peroxidase (GSH-Px) increased, and nitric oxide synthase was downregulated in the Put group (i.e., combined treatment with Put and LPS) compared with that in the LPS group (i.e., combined treatment with phosphate-buffered saline and LPS). Furthermore, activities of alkaline phosphatase and acid phosphatase were inhibited after 6 h of LPS stimulation. The expression levels of the nuclear factor kappa B, IκB kinase, Janus kinase, and signal transducer and activator of transcription proteins genes were all significantly suppressed at 12 and 24 h in the Put group. *Pseudomonas aeruginosa and Bacillus subtilis* grew better after being incubated with the serum from the Put group than that from the LPS group. Additionally, the Put treatment remarkably inhibited the autophagy of hemocytes mediated by the AMP-activated protein kinase-mammalian target of rapamycin-Beclin-1 pathway. This study demonstrated that Put can effectively inhibit the inflammatory response induced by LPS in pearl oysters. These results provide useful information for further exploration of the immunoregulatory functions of polyamines in bivalves and contribute to the development of immunosuppressive agents.

## Introduction

Many marine bivalves are economically important. However, diseases caused by bacteria or viruses present in the surrounding water environment negatively affect the development of the marine bivalve aquaculture industry (Mitta et al., 2000; Seo et al., 2010). Aside from physical and biological barriers against invaders, cellular and humoral immunity constitutes the main part of the innate immune response of bivalves (Antunes et al., 2014; Song et al., 2015). Hemocytes are an important part of the innate immune response of bivalves. Hemocytes act through phagocytosis (Hanington et al., 2010) and release cytotoxic molecules, such as nitric oxide and hydrogen, to participate in immune response (Canesi et al., 2002). Hemocytes located in areas in close contact with external microorganisms act as sentinels for any impending infection and migrate to any infection site (Allam and Espinosa, 2016). In addition, the oxidative stress caused by a bacterial infection can trigger autophagy, which is associated with cytoprotection (Yuan et al., 2009). The autophagy response of hemocytes plays a key role in the process of resisting potential pathogens in bivalves, and it determines the expression of autophagy-related genes and signaling pathways (Moreau et al., 2015; Balbi et al., 2018; Liu et al., 2020). Although hemocytes are known to have an effect on the immune system, the efficiency of defense comes from the activation of hemocytes and the components dissolved in serum, such as lectins, antioxidant enzymes, reactive oxygen species, and lysosomal enzymes (Mitta et al., 2000; Canesi et al., 2002). At the first glance, similar to that of other invertebrates, the structure of the existing immune system of bivalves is relatively simple and contains only the innate immune system, lacking an adaptive immune system (Pipe and Coles, 1995). However, accumulating evidence shows that many molecules in bivalves, including acetylcholine (Liu Z. et al., 2016; Cao et al., 2021), biogenic amines (e.g., 5-HT, epinephrine/norepinephrine, and dopamine) (Tiscar and Mosca, 2004; Dong et al., 2017), and amino acid substances (e.g., glutamic acid and γ-aminobutyric acid) (Tiscar and Mosca, 2004; Li et al., 2016), function in regulating their immune response to pathogenic bacteria, viruses, or mechanical damage.

Polyamines, such as putrescine (Put), spermidine (Spd), and spermine, are positively charged biogenic amines that are ubiquitous in all organisms, expect for certain archaeans (Chen and Martynowicz, 1984). The level of polyamines in organisms is strictly regulated. Environmental changes, tumors, oxidative stress, pathogen infection, or injury will cause changes in the polyamine levels (Wickström, 1991; Cipolla et al., 1993; Kournoutou et al., 2014). Polyamines are not only involved in cell growth, gene regulation, differentiation, and development but also have recently been found to alter the inflammatory response *in vitro* and immunity (Yuan et al., 2001; Pirinen et al., 2007; Choi and Park, 2012). They act as negative immunomodulators of natural killer cell activity (Quemener et al., 1994), lymphocytes (Byrd et al., 1977), and neutrophil locomotion (Ferrante et al., 1986). Polyamines are also important antioxidants (Guérin et al., 2001; Shoji et al., 2005; Toro-Funes et al., 2013; Liu G. et al., 2016), free radical scavengers (Ha et al., 1998), and anti-inflammatory agents (Byrd et al., 1977; Løvaas and Carlin, 1991; Li et al., 2020). In general, polyamines exert their antioxidant function by increasing the activity of antioxidant molecules of an organism, such as superoxide dismutase (SOD), catalase (CAT), and glutathione peroxidase (GSH-Px), and inhibiting the generation of free radicals (Durmu and Kadioğlu, 2005; David et al., 2008; Reyes-Becerril et al., 2011; Fang et al., 2016; Yerra et al., 2016). The immune-related genes in gilthead seabream leucocytes are actively regulated by polyamines, especially Put (Reyes-Becerril et al., 2011). Recent studies have found that many signaling pathways are involved in the regulation of the immune response of polyamines, including the nuclear factor kappa B (NF-κB), phosphoinositide 3-kinase/protein kinase B, forkhead box O-3, Janus kinase (JAK)-signal transducer and activator of transcription proteins (STAT), and mitogen-activated protein kinase pathways (Reyes-Becerril et al., 2011; Choi and Park, 2012; Li et al., 2020). Polyamines also regulate autophagy through the AMP-activated protein kinase (AMPK)-mammalian target of rapamycin (mTOR)-Unc-51 like autophagy activating kinase 1 signaling pathway (Liu et al., 2019c). In invertebrates, polyamines were first observed in *Cionia intestinalisin* in 1954 (Ackermann and Janka, 1954). Subsequently, polyamines have been detected in sea urchin, sea cucumber, sea squirt, oysters, short-necked clam, *C. elegans*, planarians, earthworms, mussel, and *Bombyx mori* (Hamana et al., 1991, 1995; Kournoutou et al., 2014; Yerra et al., 2016). In planarians and earthworms, the synthesis of Put is stimulated during regeneration, and under temperature and osmotic stresses, the levels of Put and Spd in *C. elegans*, planarians, and earthworms are temporarily increased (Hamana et al., 1995). The levels of polyamines fluctuate under Cd^+^ stress in mussels (Kournoutou et al., 2014). These results suggested that polyamines may be important regulatory molecules in stress response. However, changes in polyamines in bivalves after pathogen infection and their functions in innate immune response remain unknown.

The pearl oyster *Pinctada fucata martensii* is mainly distributed in the southern provinces of China and Japan. It is one of the most economically important species used in the production of seawater pearls. In this study, we evaluated the changes in the Put levels in pearl oysters under the LPS stress. We also investigated the immune regulation function and molecular mechanism of Put in LPS-induced inflammation. Results suggested that Put may have potential functions in oxidative stress and inflammation in pearl oysters. These results provided some data for exploring further the functions of polyamines as immunomodulators in bivalves.

## Materials and Methods

### Experimental Design and Sample Preparation

Pearl oysters (∼1.5 years old) were obtained from Houhong, Zhanjiang, Guangdong, China. The reagent was injected into the adductor muscle *via* a 100 μl microsyringe. The challenged group was injected with 100 μl of 0.5 mg/ml LPS (Sigma-Aldrich, St. Louis, MO, United States). The control group was injected with 100 μl of 1 × phosphate-buffered saline (1 × PBS) in the same way. In the Put group, 1 h before LPS injection, 100 μl of 1 mM Put dihydrochloride (Sigma-Aldrich, St. Louis, MO, United States) was injected. The pearl oysters injected with 100 μl of the 1 × PBS solution were used as the control group before LPS stimulation (i.e., LPS group). After LPS stimulation, the pearl oysters were maintained at (25 ± 1)^°^C in cistern with filtered seawater. Hemolymph was then extracted from eight pearl oysters in each group by using a 2 ml syringe at 6, 12, 24, and 48 h after LPS stimulation. Hemocytes and serum were separated from the hemolymph *via* centrifugation at 3,000 r/min for 5 min at 4°C. The collected hemocytes were resuspended in the TRIzol reagent (Invitrogen, Carlsbad, CA, United States). Both serum and hemocytes were quick-frozen in liquid nitrogen and stored at −80°C until use.

### Detection of Put Content in Serum After Lipopolysaccharide Stimulation

Serum was collected from five individuals at different times post-LPS stimulation in each group. The Put levels were determined using kits (mlbio, Shanghai, China) following the instructions of the manufacturer. The procedure includes the following: 10 μl of the testing sample, 40 μl of the sample diluent, and 100 μl of the horseradish peroxidase-conjugate reagent were added to each well. Then, it was covered with an adhesive strip and incubated for 60 min at 37°C. Each well was aspirated and washed, repeating the process four times for a total of five washes. Notably, 50 μl of chromogen solution A and 50 μl of chromogen solution B were added to each well, gently mixed, incubated for 15 min at 37°C, and protected from light. Finally, 50 μl of stop solution was added to each well, and the OD450 value of each well at 37°C was determined using a microplate reader. Five samples of parallel tests were performed on each group.

### Detection of Immune-Related Enzymes and Antibacterial Activities of Serum

After the Put treatment, the activities of antioxidant-related enzymes were detected in five individuals. SOD, CAT, activities of lysozyme (LYS), acid phosphatase (ACP), alkaline phosphatase (ALP), and GSH-Px were detected using kits (mlbio, Shanghai, China) in accordance with the instructions. The detection protocols are the same as the detection protocols of the Put level described in the “Detection of Put content in serum after LPS stimulation” section. The activity of iNOS was tested by kits (Nanjing Jiancheng Bioengineering Research Institute, Nanjing, China) following the protocol of the manufacturer. First, 30 μl serum and 40 μl reagent 4 were mixed. Then, 200 μl reagent 1, 10 μl reagent 2, and 100 μl reagent 3 were added to each tube. They were gently mixed and incubated at 37°C for 15 min. Finally, 100 μl of reagent 4 and 2 ml of reagent 5 were added, and the OD450 value was detected using a microplate reader.

The antibacterial activity of serum after the Put treatment was detected. *Pseudomonas aeruginosa* and *Bacillus subtilis* were cultured to the logarithmic growth phase with the LB medium. Afterward, 10 μl of the bacterial solution and 50 μl of serum were added to a sterile 96-well plate and mixed well. After incubating for 3 h at room temperature, 200 μl of LB medium was added to each well. The OD600 value of each well at 37°C was determined using a microplate reader (the test was performed once every 30 min and continued for 24 h). Five samples of parallel tests were performed on each group.

### Real-Time PCR for Detection of the Expression of Immune-Related Genes After Put Treatment

The total RNA of all samples was isolated using the TRIzol method (Invitrogen, United States). The cDNA template was prepared with Moloney murine leukemia virus (M-MLV) reverse transcriptase (RT) (Takara, Clontech, Japan). The relative expression of target genes was detected *via* quantitative RT-PCR (qRT-PCR) performed on the Applied Biosystems 7500/7500 instrument. β-Actin was selected to assess the relative expression of genes. All the primers used herein were designed using the Primer Premier 5 software (Table 1).

**TABLE 1:** Primer sequences used in this study.

Primer name	Primer sequence (5′-3′)
NF-κB-F	AGAAGAGACAGGCCAAAGAGCA
NF-κB-R	AGAGAGAACAGGCGTGAGAAGC
Ikk-F	TATTAAAGGCTCAGGCAGAGGTAT
Ikk-R	TTGGAGTTGCTGATTACGGATT
STAT-F	TTTCAAGATTCACAAGCCCAACT
STAT-R	AACTTTCCCATTTCCTCCCG
JAK-F	ATGGAGTTATGGCGTTCTTATGTG
JAK-R	ATGCTGCTTTGGCTGTTTCG
β-actin-F	CGGTACCACCATGTTCTCAG
β-actin-R	GACCGGATTCATCGTATTCC
mTOR-F	CCGTATGGAAGCGGTCAGAAC
mTOR-R	TGTGATGCCCACGACCAGTAGT
Beclin-1-F	TCTAACGCTCCTTTGATTCCACA
Beclin-1-R	CTTGCTACCTTGACCCTATGACTGA
AMPK-F	ATTCTTGGCGATACTCTGGGTGT
AMPK-R	CCCTGCTACATATTCCATCACCA

*AMPK, AMP-activated protein kinase; IKK, IκB kinase; JAK, Janus kinase; mTOR, mammalian target of rapamycin; NF-κB, nuclear factor kappa B; STAT, signal transducer and activator of transcription proteins.*

### Monodansylcadaverine Assay for Autophagy Evaluation

The autophagy levels in hemocytes were detected using the monodansylcadaverine (MDC) autophagy staining detection kit (Beijing Solarbio Science & Technology, Beijing, China). After 48 h of Put + LPS treatment, 200 μl of hemolymph was drawn using a 2 ml syringe, and then the hemolymph was spread on the cover glass. After 10 min, the cells were washed twice with 1× washing buffer and the supernatant was removed. Then, 90 μl of 1× washing buffer and 10 μl of MDC stain were added and the cells were incubated at room temperature for 1 h. Three samples of parallel tests were performed on each group. The hemocyte autophagy levels were observed and photographed using a fluorescence microscope (Nikon ECLIPSE Ni, DS-Ri2, Tokyo, Japan). Five fields of view were randomly selected for each sample to calculate the autophagy ratio of hemocytes.

### Data Analysis

The data on qRT-PCR, detection of enzyme activity, and antibacterial activity were analyzed using the SPSS 22.2 software. Differences in enzyme activity and gene expression level at different time points in the same group were determined using ANOVA. Differences in enzyme activity, gene expression level, bacterial growth level, and hemocyte autophagy levels between different groups at the same time point were determined *via t*-test. *P*-values < 0.05 were considered statistically significant.

## Results

### Changes in Put Concentration in Serum After Lipopolysaccharide Stimulation

**FIGURE 1:**
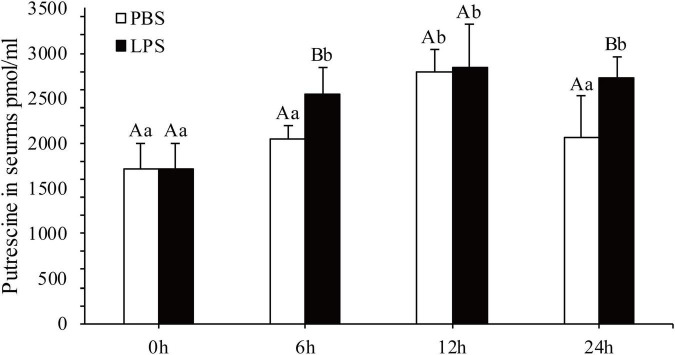
Detection of the putrescine level in serum after lipopolysaccharide (LPS) stimulation. Values are shown as mean ± SE (*N* = 5). Lowercase letters represent differences at different time points in the same groups, and capital letters represent differences in the different groups at same time points.

The concentration of Put in serum after 6, 12, and 24 h of LPS stimulation was quantified (Figure 1). In contrast to that in the in the PBS group, Put concentrations in serum significantly increased after 6 and 24 h by 1.24- and 1.32-fold, respectively, in the LPS stimulation group (*P* < 0.05). However, changes in Put concentrations in serum were not significant after 12 h of LPS stimulation (*P* > 0.05). Therefore, the Put concentration was sensitive to LPS stimulation in pearl oysters.

### Immune- and Antioxidant-Related Enzymes and Antibacterial Activities

**FIGURE 2:**
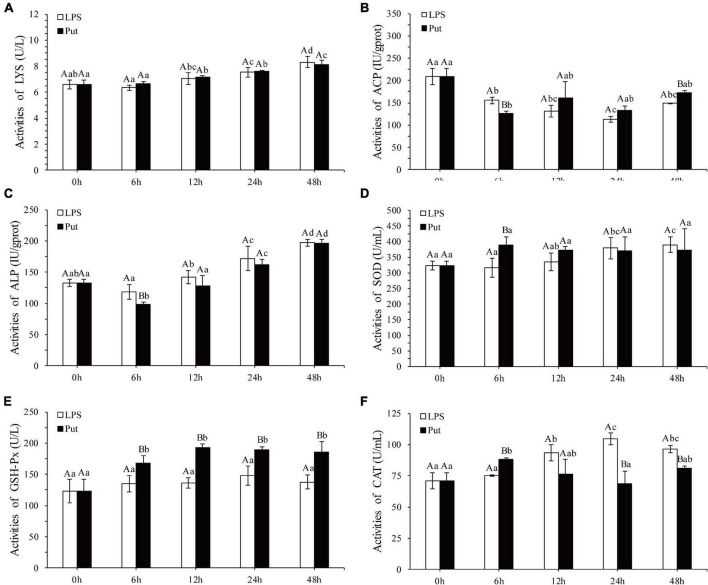
Detection of enzyme activity after the putrescine treatment. LYS, lysozyme; ACP, acid phosphatase; ALP, alkaline phosphatase; SOD, superoxide dismutase; GSH-Px, glutathione peroxidase; CAT, catalase. Values are shown as mean ± SE (*N* = 5). Lowercase letters represent differences at different time points in the same groups, and capital letters represent differences in the different groups at same time points. Lipopolysaccharide (LPS) group, PBS_LPS; Put group, Put dihydrochloride_LPS. **(A–F)** represent the activities of LYS/ACP/ALP/SOD/GSH-Px/CAT after the putrescine treatment, respectively.

**FIGURE 3:**
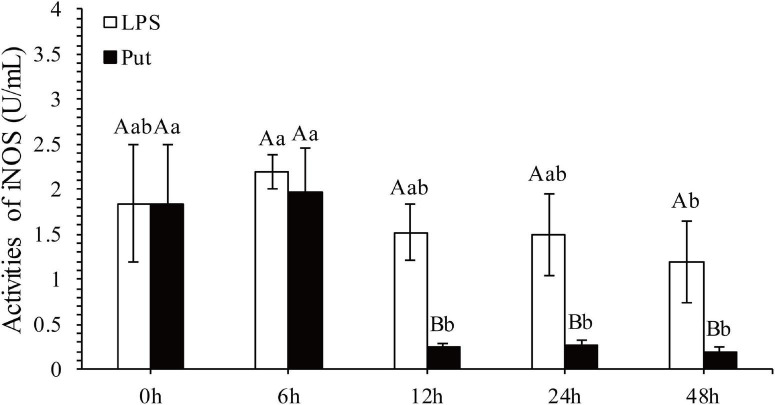
Detection of iNOS activity in serum after the putrescine treatment. Values are shown as mean ± SE (*N* = 5). iNOS, inducible nitric oxide synthase. Lowercase letters represent differences at different time points in the same groups, and capital letters represent differences in the different groups at same time points. Lipopolysaccharide (LPS) group, PBS_LPS; Put group, Put dihydrochloride_LPS.

The regulatory functions of Put in LPS-induced humoral immunity were investigated by determining the LYS, ACP, ALP, SOD, CAT, GSH-Px, and inducible nitric oxide synthase (iNOS) in the Put group (i.e., combined treatment with Put and LPS) and the LPS group (i.e., combined treatment with PBS and LPS). As shown in Figures 2A–C, ACP and ALP activities were significantly inhibited by 1.24- and 1.21-fold, respectively, at 6 h in the Put group. However, the ACP activity was significantly induced at 48 h in the Put group compared with that in the LPS group (Figures 2B,C, *P* < 0.05). In contrast, the LYS activity did not significantly change (Figure 2A, *P* > 0.05). Furthermore, the SOD activity increased by 1.23-fold at 6 h in the Put group compared with that in the LPS group (Figure 2D, *P* < 0.05). In the Put group, the GSH-Px activity significantly increased by 1.25- to 1.42-fold at 6–48 h compared with that in the LPS group (Figure 2E, *P* < 0.05). The CAT activity was upregulated by 1.17-fold at 6 h and then downregulated at 24 and 48 h in the Put group compared with that in the LPS group (Figure 2F, *P* < 0.05). The iNOS activity was downregulated by 5.45- to 6.1-fold at 12–48 h in the Put group compared with that in the LPS group (Figure 3, *P* < 0.05). These results indicated that Put pretreatment remarkably attenuated oxidative stress.

**FIGURE 4:**
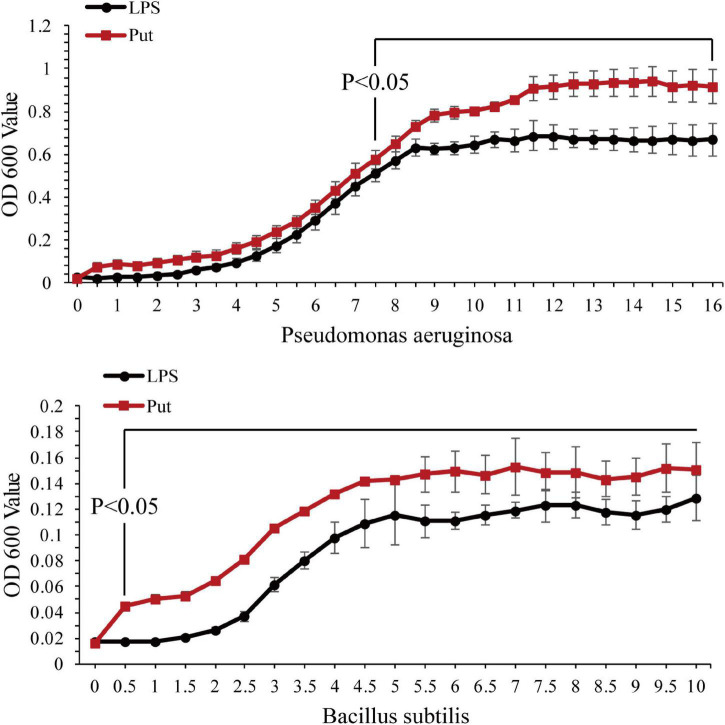
Antibacterial activity of serum after the putrescine treatment. Values are shown as mean ± SE (*N* = 5), and different superscripts indicate significant differences at *P* < 0.05. Lipopolysaccharide (LPS) group, PBS_LPS; Put group, Put dihydrochloride_LPS. Lowercase letters represent differences at different time points in the same groups, and capital letters represent differences in the different groups at same time points.

The antibacterial activity of serum was determined using a microplate reader. As shown in Figure 4, *P. aeruginosa* and *B. subtilis* grew significantly better after co-incubation with the serum from the Put group at 48 h than that with the serum from the LPS group (*P* < 0.05, Figure 4).

### Changes in Expression of Immune-Related Genes

**FIGURE 5:**
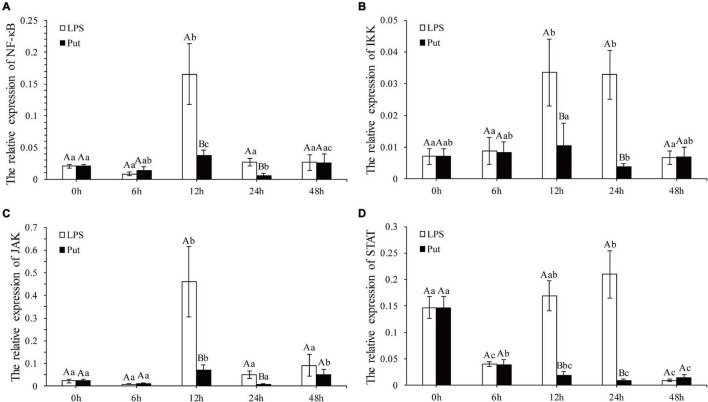
Expression of the nuclear factor kappa B (NF-κB), IκB kinase (IKK), Janus kinase (JAK), and signal transducer and activator of transcription proteins (STAT) genes after putrescine treatment. Values are shown as mean ± SE (*N* = 5). Lipopolysaccharide (LPS) group, PBS_LPS; Put group, Put dihydrochloride_LPS. Lowercase letters represent differences at different time points in the same groups, and capital letters represent differences in the different groups at same time points. **(A–D)** represent the expression of NF-κB/IKK/JAK/STAT after the putrescine treatment, respectively.

The NF-K B/JAK-STAT signaling pathway is a potential pathway to mediate the oxidative stress and inflammation. The expression levels of NF-K B, IKK, JAK, and STAT genes were determined *via* qRT-PCR (Figure 5). Compared with those in the LPS group, the expression levels of NF-κB and IKK from the NF-κB signaling pathway were significantly downregulated at 12 and 24 h by 3.16- to 8.23-fold in the Put group, respectively (*P* < 0.05, Figures 5A,B). The relative expressions of two genes from the JAK/STAT signaling pathway investigated herein were all significantly suppressed, declining by approximately 6.3- to 24.56-fold for JAK and STAT at 12 and 24 h, respectively (*P* < 0.05, Figures 5C,D).

### Detection of Hemocyte Autophagy Level

**FIGURE 6:**
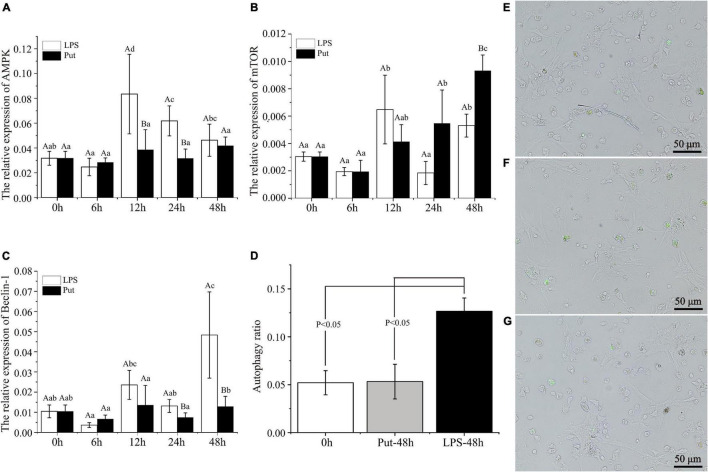
Putrescine treatment significantly inhibited hemocyte autophagy in pearl oysters. **(A–C)** mRNA expression of AMP-activated protein kinase (AMPK), mammalian target of rapamycin (mTOR), and Beclin-1 in hemocytes was analyzed. Values are shown as mean ± SE (*N* = 5). Lowercase letters represent differences at different time points in the same groups, and capital letters represent differences in the different groups at same time points. **(D–G)** Level of autophagy in hemocytes was detected *via* the monodansylcadaverine (MDC) method. Green fluorescence represents a positive signal. Lipopolysaccharide (LPS) group, PBS_LPS; Put group, Put dihydrochloride_LPS.

To achieve a better understanding of the underlying molecular mechanisms of Put-mediated autophagy inhibition, we explored the possible involvement of signaling pathways. Compared with the 0 h group, AMPK was significantly upregulated by 2.51- to 2.63-fold at 12 and 24 h by LPS stimulation, respectively (*P* < 0.05, Figure 6A). Moreover, mTOR was highly induced by 2.13- and 1.74-fold at 12 and 48 h, respectively (*P* < 0.05, Figure 6B). In the LPS group, Beclin-1 was highly induced by 4.63-fold at 48 h compared with 0 h (*P* < 0.05, Figure 6C). These results showed that autophagy was induced by LPS stimulation. Compared with that in the LPS group, the expression of AMPK was obviously decreased by 1.77- and 3.75-fold at 12 and 24 h, respectively, whereas that of mTOR increased by 1.75-fold at 48 h in the Put group (*P* < 0.05, Figures 6A,B). Compared with that in the LPS group, Beclin-1 expression decreased to 1.77- and 3.75-fold at 24 and 48 h, respectively, in the Put group (*P* < 0.05, Figure 6C).

Subsequently, we adopted the MDC method to detect the autophagy level of pearl oyster hemocytes (Figures 6D–G). In the LPS group, the hemocyte autophagy level increased from 5.2 to 12.7% compared with that in the 0 h group (*P* < 0.05). Compared with that in the LPS group, the hemocyte autophagy level decreased from 12.7 to 5.3% at 48 h in the Put group (*P* < 0.05). Therefore, the Put pretreatment inhibited hemocytes autophagy *via* the AMPK-mTOR-Beclin-1 pathway upon LPS stimulation.

## Discussion

Polyamines are a class of ubiquitous positively charged biogenic amines (Bae et al., 2018). In vertebrates, polyamines are important antioxidants and anti-inflammatory agents, and they regulate the immune response (Guérin et al., 2001; Shoji et al., 2005; Rider et al., 2007; Toro-Funes et al., 2013; Liu G. et al., 2016). Polyamines are also widespread in bivalve mollusks, suggesting that they may also be an important immune regulatory molecule (Hamana et al., 1991; Kournoutou et al., 2014). However, the immunomodulatory function of polyamines in the immune response of bivalves remains unknown. In this study, Put accumulation occurred at 6 and 24 h after LPS stimulation in pearl oysters. In eukaryotes, changes in the polyamine levels occur under various stresses, such as oxidative stress, pathogen infection, tissue damage, and drought/temperature stress (Wickström, 1991; Cipolla et al., 1993; Kournoutou et al., 2014; Adamipour et al., 2020; Tsaniklidis et al., 2021). In mussels, an increase in Put concentration under Cd^+^ stress may play an important role in the oxidative stress caused by this cation (Kournoutou et al., 2014). Polyamine accumulation and extensive changes in its levels under stress conditions indicate that they play a role in adaptive responses to various environmental stresses. Therefore, the accumulation of Put after LPS stimulation in pearl oysters indicated that it may play an important role in LPS-induced immune response.

To explore further the function of Put in LPS stress, we used this polyamine to treat pearl oysters before LPS stimulation. Owing to their lack of adaptive immunity, pearl oysters mainly rely on hemocytes and humoral immunity to resist pathogenic invasion (Pipe and Coles, 1995; Antunes et al., 2014; Song et al., 2015). LYS, ACP, and ALP are important antibacterial molecules in the humoral immunity of bivalves. In this study, the activities of ACP and ALP in pearl oyster were considerably inhibited in the Put group, which were consistent with those reported in mammals (Jing et al., 2021). Bacterial infections can cause oxidative stress. SOD, CAT, and GSH-Px are the main parameters of oxidative stress (Bilbao et al., 2009; Kolaiti et al., 2009; Venier et al., 2011). The increased activity of the antioxidant-related enzyme observed in our research suggested that oxidative stress weakened when treated with Put. In mammals, diets supplemented with Put substantially increase the activities of SOD, CAT, and GSH-Px (Liu et al., 2019a). Reactive nitrogen species, such as NO, are also free radicals that cause oxidative stress. iNOS is a critical enzyme that catalyzes the biosynthesis of NO. Our results showed that the iNOS activity was effectively inhibited after the Put treatment, similar to that observed in vertebrates (Choi and Park, 2012; Liu et al., 2019b). These results indicate that Put can effectively attenuate the oxidative stress response caused by LPS stimulation. Furthermore, the expression levels of NF-κB, IKK, JAK, and STAT genes were notably downregulated in hemocytes to be pretreated with Put before LPS stimulation. Previous studies have shown that inhibiting the NF-κB/JAK-STAT signaling pathway can remarkably reduce the oxidative stress and inflammation response (Choi and Park, 2012; Zhao et al., 2017; Liu et al., 2019b; Hu et al., 2020). Therefore, we speculated that Put may inhibit the LPS-stimulated oxidative stress by blocking the NF-κB and the JAK/STAT signaling pathways in the hemocytes of pearl oysters.

Autophagy is one of the main sensors of the oxidative stress signal (Lee et al., 2012). To respond to bacterial infections and prevent damage caused by oxidative stress, cells activate their autophagy system (Nakagawa et al., 2004; Travassos et al., 2010; Lee et al., 2012). In this study, the hemocyte autophagy level of pearl oysters decreased after the Put treatment. In vertebrates, the inhibition of iNOS by drug inhibitors can reduce autophagy in cardiomyocytes (Yuan et al., 2009). Therefore, the inhibition of the iNOS activity by the Put treatment may be one of the reasons for the decrease in hemocyte autophagy. Numerous key genes and signaling pathways involved in autophagy have been identified, such as mTOR, AMPK, and Beclin-1 (He and Klionsky, 2009; Booth et al., 2018). Autophagy-related genes are regulated by mTOR (Rosenbluth and Pietenpol, 2009). Inhibition of mTOR activity induces autophagy (Floto et al., 2007). Beclin-1 is a crucial protein that initiates autophagy (Booth et al., 2018). AMPK is an energy sensor that mediates autophagy (Harhaji-Trajkovic et al., 2009). Furthermore, it has been revealed that polyamines regulate autophagy through the AMPK-mTOR pathway (Pichiah et al., 2011; Liu et al., 2019c; Chen et al., 2021). To better understand the molecular mechanism of autophagy regulated by Put, we explored the possible involvement of signaling pathways. Results showed that Put inhibited the expression of AMPK and Beclin genes and induced that of mTOR, suggesting that Put may inhibit LPS-induced hemocyte autophagy through the AMPK-mTOR-Beclin-1 pathway.

## Conclusion

The Put levels were induced after LPS stimulation in pearl oysters. Put weakened the oxidative stress in pearl oysters. The NF-κB/JAK-STAT pathways regulated by Put may be the potential pathways that regulate oxidative stress. Put inhibited hemocyte autophagy through the AMPK-mTOR-Beclin-1 pathway. Pearl oyster is one of the most economically important species for cultivating seawater pearls. During artificial pearl breeding, the damage and pathogenic infection caused during transplantation will cause strong immune rejection and even the death of pearl oysters. Therefore, the function of Put as an immunomodulator in pearl cultivation will be explored in a follow-up study.

## Data Availability Statement

The original contributions presented in the study are included in the article/supplementary material, further inquiries can be directed to the corresponding author.

## Ethics Statement

The pearl oyster *Pinctada fucata martensii* is a lower invertebrate, and therefore, the study was not subject to ethical approval.

## Author Contributions

YJ, XD, and ZZ: conceptualization. YC: writing—original draft preparation. SZ, XL, ZL, and JC: validation. XX and ZG: formal analysis. ZZ and YJ: writing—review and editing. All authors have read and agreed to the published version of the manuscript.

## Conflict of Interest

The authors declare that the research was conducted in the absence of any commercial or financial relationships that could be construed as a potential conflict of interest.

## Publisher’s Note

All claims expressed in this article are solely those of the authors and do not necessarily represent those of their affiliated organizations, or those of the publisher, the editors and the reviewers. Any product that may be evaluated in this article, or claim that may be made by its manufacturer, is not guaranteed or endorsed by the publisher.
